# Effects of the Endocrine-Disrupting Chemical DDT on Self-Renewal and Differentiation of Human Mesenchymal Stem Cells

**DOI:** 10.1289/ehp.1408188

**Published:** 2014-07-11

**Authors:** Amy L. Strong, Zhenzhen Shi, Michael J. Strong, David F.B. Miller, Douglas B. Rusch, Aaron M. Buechlein, Erik K. Flemington, John A. McLachlan, Kenneth P. Nephew, Matthew E. Burow, Bruce A. Bunnell

**Affiliations:** 1 Center for Stem Cell Research and Regenerative Medicine, and; 2 Department of Pathology, Tulane University School of Medicine, New Orleans, Louisiana, USA; 3 Tulane Cancer Center, Tulane Health Science Center, New Orleans, Louisiana, USA; 4 Medical Sciences, and; 5 Department of Cellular and Integrative Physiology, Indiana University, Bloomington, School of Medicine, Bloomington, Indiana, USA; 6 Center for Genomics and Bioinformatics, Indiana University, Bloomington, Bloomington, Indiana, USA; 7 Department of Pharmacology, Tulane University School of Medicine, New Orleans, Louisiana, USA; 8 Department of Medicine, Section of Hematology and Medical Oncology, Tulane Health Sciences Center, New Orleans, Louisiana, USA

## Abstract

Background: Although the global use of the endocrine-disrupting chemical DDT has decreased, its persistence in the environment has resulted in continued human exposure. Accumulating evidence suggests that DDT exposure has long-term adverse effects on development, yet the impact on growth and differentiation of adult stem cells remains unclear.

Objectives: Human mesenchymal stem cells (MSCs) exposed to DDT were used to evaluate the impact on stem cell biology.

Methods: We assessed DDT-treated MSCs for self-renewal, proliferation, and differentiation potential. Whole genome RNA sequencing was performed to assess gene expression in DDT-treated MSCs.

Results: MSCs exposed to DDT formed fewer colonies, suggesting a reduction in self-renewal potential. DDT enhanced both adipogenic and osteogenic differentiation, which was confirmed by increased mRNA expression of glucose transporter type 4 (*GLUT4*), lipoprotein lipase (*LpL*), peroxisome proliferator-activated receptor gamma (*PPAR*γ), leptin, osteonectin, core binding factor 1 (*CBFA1*), and FBJ murine osteosarcoma viral oncogene homolog (*c-Fos*). Expression of factors in DDT-treated cells was similar to that in estrogen-treated MSCs, suggesting that DDT may function via the estrogen receptor (ER)-mediated pathway. The coadministration of ICI 182,780 blocked the effects of DDT. RNA sequencing revealed 121 genes and noncoding RNAs to be differentially expressed in DDT-treated MSCs compared with controls cells.

Conclusion: Human MSCs provide a powerful biological system to investigate and identify the molecular mechanisms underlying the effects of environmental agents on stem cells and human health. MSCs exposed to DDT demonstrated profound alterations in self-renewal, proliferation, differentiation, and gene expression, which may partially explain the homeostatic imbalance and increased cancer incidence among those exposed to long-term EDCs.

Citation: Strong AL, Shi Z, Strong MJ, Miller DF, Rusch DB, Buechlein AM, Flemington EK, McLachlan JA, Nephew KP, Burow ME, Bunnell BA. 2015. Effects of the endocrine-disrupting chemical DDT on self-renewal and differentiation of human mesenchymal stem cells. Environ Health Perspect 123:42–48; http://dx.doi.org/10.1289/ehp.1408188

## Introduction

Endocrine-disrupting chemicals (EDCs) are natural or synthetic compounds capable of altering hormonal and homeostatic systems. Although factors such as age, duration, EDC type, and dose–response dynamics influence the severity of EDC-driven changes, these chemicals remain a cause for concern because of their similarity to endogenous hormones and their induction of downstream signaling cascades (Diamanti-Kandarakis et al. 2009). Evidence suggests that epigenetic modifications associated with EDC exposure may play a role in adverse outcomes both early in development and within stem cells in adult tissues (Crews and McLachlan 2006). Thus, mechanistic studies that explore the impact of EDCs on stem cell biology and fate are critical.

DDT (dichlorodiphenyltrichloroethane) is an EDC whose effects on estrogen receptor (ER) function are well documented. DDT is a widespread environmental contaminant as a result of its prevalent use in both agriculture and malaria control. DDT is known to negatively influence reproductive development via disruption of multiple endocrine pathways (Holm et al. 2006). Studies have reported that DDT disrupts both male and female reproductive organs. For example, in female rats exposed to DDT, high levels of gonadotropin-releasing hormone resulted in premature development of the reproductive system (Di Gregorio et al. 2001; Rasier et al. 2007, 2008).

DDT levels in breast milk have been reported to exceed the tolerable daily intake and maximum residue limit in dairy milk consumed by adults, thereby increasing the exposure of infants to DDT through breast feeding (Bouwman et al. 2006; Okonkwo et al. 2008). In developing countries, rates of urogenital malformations in male infants were elevated among those exposed to DDT (Bornman et al. 2010). The continuous exposure to DDT throughout childhood and adolescence has been reported to result in significant reproductive abnormalities, germ line cancers, sexual precocity, early puberty, and reduced pregnancy rates in females (Cohn et al. 2003; Krstevska-Konstantinova et al. 2001). Several studies have indicated an inverse correlation between DDT and blood hormone levels, resulting in low infant birth weight, cryptorchidism, and decreased sperm motility and semen quality in males (Ben Rhouma et al. 2001; Bhatia et al. 2005; Longnecker et al. 2002). Although the precise mechanism by which DDT influences germ line cells is unknown, DDT and related organochlorine pesticides have long been know to mimic estrogens (Bulger and Kupfer 1983). Recently, DDT has been shown to exert estrogenic effects through a variety of molecular mechanisms involving ERα and other cellular signaling systems, such as AP-1 (activator protein 1), p38, and ERK1/2 (extracellular signal-regulated protein kinases 1 and 2) (Bratton et al. 2012; Frigo et al. 2005; Han et al. 2008; McLachlan et al. 2012). Although it has been established that DDT exposure alters both cellular and molecular phenotypes in humans, strikingly little is understood about its effects on tissue-resident stem cells. Mesenchymal stem cells (MSCs) in the bone marrow are directly involved in hematopoietic stem cell maturation and migration into the circulation, and they are key regulators of the immune system (Auletta et al. 2012). Because of their immunomodulatory and regenerative potential, MSCs are currently being investigated and used clinically to treat autoimmune diseases and regenerate bone and cartilage (Granero-Molto et al. 2008; Yamout et al. 2010). MSCs undergo self-renewal and can be efficiently differentiated into bone, adipose, and cartilage tissue. Understanding the impact of EDCs on the efficiency of MSC self-renewal, proliferation, and differentiation will address whether EDC exposure negatively affects their ability to function normally *in vivo* and their potential use in regenerative medicine. In the present study, we used human MSCs to investigate the mechanism(s) of action of DDT. MSCs were exposed to DDT, and the impact on morphology, self-renewal, proliferation, differentiation, and gene expression profiles was examined. The results show that DDT-treated MSCs exhibited profound alterations in these essential biological properties and indicated that these altered processes may reflect homeostatic imbalance and increased cancer incidence among those exposed to EDCs.

## Materials and Methods

*Chemicals*. We purchased DDT from AccuStandard (New Haven, CT) and 17β-estradiol (E_2_), fulvestrant [ICI 182,780 (ICI)], dexamethasone, isobuytlmethylxanthine, indomethacin, ascorbate 2-phosphate, β-glycerol phosphate, Oil Red O, Alizarin Red S, cetylpyridinium chloride, and crystal violet from Sigma-Aldrich (St. Louis, MO).

*Isolation of MSCs*. Under a protocol approved by the Tulane University Institutional Review Board, we obtained MSCs from three normal healthy donors who provided informed consent. The cells were prepared from bone marrow aspirates using standard protocols, as previously described (Strong et al. 2013a). Briefly, bone marrow aspirates were taken from the iliac crest of normal adult donors. Cells were isolated using a density gradient (Ficoll-Paque; Amersham Pharmacia Biotech, Milwaukee, WI) and cultured in complete culture media (CCM), composed of α-MEM (Minimum Essential Medium α; GIBCO, Life Technologies, Grand Island, NY), 20% fetal bovine serum (FBS; Atlanta Biologicals, Lawrenceville, GA), 100 U/mL penicillin/100 μg/mL streptomycin (GIBCO), and 2 mM l-glutamine (GIBCO). When the cultures reached 70% confluency, the cells were harvested with 0.25% trypsin/1 mM EDTA, resuspended at 1 × 10^6^ cells/mL in α-MEM with 5% dimethyl sulfoxide (DMSO) and 30% FBS, frozen in 1-mL aliquots overnight at –80^o^C, and stored in liquid nitrogen for no more than 6 months before thawing. Donor demographic information is available in Supplemental Material, Table S1.

*Cell culture*. MSCs (10^6^) were thawed, plated in CCM, and incubated at 37°C with 5% humidified CO_2_. After 24 hr, viable cells were washed, harvested with 0.25% trypsin/1 mM EDTA, and replated at 100 cells/cm^2^ in CCM. Medium was changed every 3–4 days. For all experiments, we used subconfluent cells (≤ 70% confluent) between passages 2 and 6. For some experiments, MSCs from each donor were treated separately with DMSO (vehicle control), 10 nM E_2_, or 1 μM DDT for 5 days in α-MEM supplemented with 20% charcoal-dextrose–stripped FBS (CDS-FBS), 2 mM l-glutamine, and penicillin/streptomycin. To investigate the dose–response relationship, we treated MSCs with DDT at 100 pM, 1 nM, 10 nM, 100 nM, 1 μM, or 10 μM. For other experiments, combined treatment of 10 nM E_2_ + 100 nM ICI or 1 μM DDT + 100 nM ICI was administered for 5 days. Images of cultured cells were acquired using a Nikon Eclipse TE200 microscope, a DXM1200F digital camera, and ACT-1 software, version 2.7 (all from Nikon, Melville, NY).

MCF7 cells were obtained from ATCC (Manassas, VA) and cultured in Dulbecco’s modified Eagle’s medium (DMEM; GIBCO), supplemented with 10% FBS and penicillin/streptomycin. Cells were grown at 37°C with 5% humidified CO_2_, fed every 2–3 days, and split 1:4 when they reached 90% confluency.

*Colony-forming unit (CFU) assay*. MSCs were plated in triplicate at 1.8 cells/cm^2^ in 6-well plates (Nunc). After 14 days in culture, plates were washed with phosphate-buffered saline (PBS) and cells were stained with 3% crystal violet for 30 min. Plates were then washed and colonies (≤ 2 mm^2^ in diameter) were counted. After counting, plates were destained with methanol, and absorbance values were measured at 584 nm using a FLUOstar OPTIMA microplate reader (BMG Labtech Inc., Durham, NC). Values represent the ratio of the absorbance value to the number of CFUs and are given as arbitrary units (AU). Assays were performed three independent times.

*MTT assay*. Cell viability was assessed using the MTT [3-(4,5-dimethylthiazol-2-yl)-2,5-diphenyltetrazolium bromide] assay. MSCs were plated in triplicate in 96-well plates (500 cells/well). After 1, 2, 4, or 7 days, cells were incubated with 10 mM MTT (Invitrogen; Grand Island, NY) for 4 hr at 37°C with 5% humidified CO_2_. A total of 100 μL of dissolving solution (10% SDS, 0.01 M HCl) was added to each well and incubated for 12–16 hr at 37°C. Absorbance was measured at 544 nm (FLUOstar Optima). Each experiment was performed three independent times.

*Differentiation protocols*. MSCs (10^5^) from each donor were plated in 6-well plates in triplicate and cultured in CCM until reaching 70% confluence. MSCs were then incubated with DMSO vehicle, E_2_, or DDT for 5 days. To examine adipogenic differentiation, we replaced CCM with media containing adipogenic supplements: 0.5 μM dexamethasone, 0.5 mM isobuytlmethylxanthine, and 50 μM indomethacin. For osteogenic differentiation, CCM was replaced with media containing osteogenic supplements: 50 μM ascorbate 2-phosphate, 10 mM β-glycerol phosphate, and 10 nM dexamethasone. After 2 weeks, cells were fixed, washed, and stained with 0.5% Oil Red O (adipogenic differentiation) or 40 mM Alizarin Red S (osteogenic differentiation). Images were acquired as described above.

For quantification of differentiation, Oil Red O and Alizarin Red S were extracted from each well with isopropanol and 10% cetylpyridinium chloride, respectively, and read at 584 nm (FLUOstar OPTIMA). To normalize values to the amount of protein in each sample, we performed protein extraction using RIPA buffer and protein quantification using the BCA assay (both from Thermo Scientific, Waltham, MA) according to manufacturer’s instructions. Experiments were conducted three independent times.

*RNA isolation and quantitative reverse transcription polymerase chain reaction (qRT-PCR)*. MSCs (10^6^) from each donor (*n* = 3) and MCF7 cells were treated separately with DMSO vehicle, 1 nM estrogen, or 1 μM DDT for 5 days. Total cellular RNA was extracted from MSCs using TRIzol reagent, purified with DNase I digestion, and reverse transcribed using the SuperScript VILO cDNA synthesis kit (all from Invitrogen). PCR was performed in triplicate using the EXPRESS SYBR GreenER qPCR SuperMix Kit (Invitrogen) according to the manufacturer’s instructions. Primer sequences are provided in Supplemental Material, Table S2. The expression of human β-*actin* was used to normalize mRNA content.

*Sample preparation, cDNA library preparation, and next-generation sequencing*. MSCs (10^6^) were treated with 1 μM DDT or vehicle. After 5 days, cells were trypsinized, washed with PBS, and stored at –80^o^C before RNA isolation. We performed RNA sequencing (RNA-seq) as previously described by Miller et al. (2013). cDNA libraries were prepared from ribo-depleted total RNA and sequenced on an Illumina HiSeq 2000 instrument (Illumina, San Diego, CA).

*Gene expression and hierarchical clustering analysis*. Before RNA-seq reads were aligned to the human genome, three nucleotides from the 5´ end of each read were trimmed using PrinSeq (default options; http://prinseq.sourceforge.net/). Reads were then aligned to the human genome (hg19; UCSC Genome Bioinformatics; http://genome.ucsc.edu/) using Tophat V2.0.8 (default options) (Trapnell et al. 2009). Transcript data from Tophat were analyzed using HTseq (-m intersection-strict; default options) (Anders 2010) for transcript quantification of human genes along with noncoding RNAs. The transcript counts were then imported into the R software environment and analyzed using the edgeR package (Robinson et al. 2010). Following adjustment for multiple testing (Benjamini-Hochberg correction), the threshold for significant differential expression was set at *p* < 0.05. Genes that were significantly differentially expressed between vehicle- and DDT-treated MSCs were clustered using the Pearson and Spearman correlation coefficient and complete linkage algorithms in order to detect groups of coexpressed genes. Ingenuity Pathway Analysis software (Ingenuity Systems; Redwood City, CA) was used to identify relevant pathways altered by DDT, based on differentially expressed genes.

*Statistical analysis*. All values are presented as mean ± SD. Experiments were conducted in triplicate, using samples from three independent donors, with the three biological replicates performed at separate times. We calculated the average of the triplicates for each donor per replicate, and then calculated the average for each replicate (independent trial) with respect to each donor. Finally, we calculated the mean ± SD for the three donors combined. The statistical differences between three or more groups were determined by analysis of variance, followed by post hoc Dunnett multiple comparison tests, compared with the respective control group. The statistical differences between two groups were performed by Student’s *t*-test. Statistical significance was set at *p* < 0.05. Analyses were performed using GraphPad Prism (GraphPad Software, San Diego, CA).

## Results

**Figure 1:**
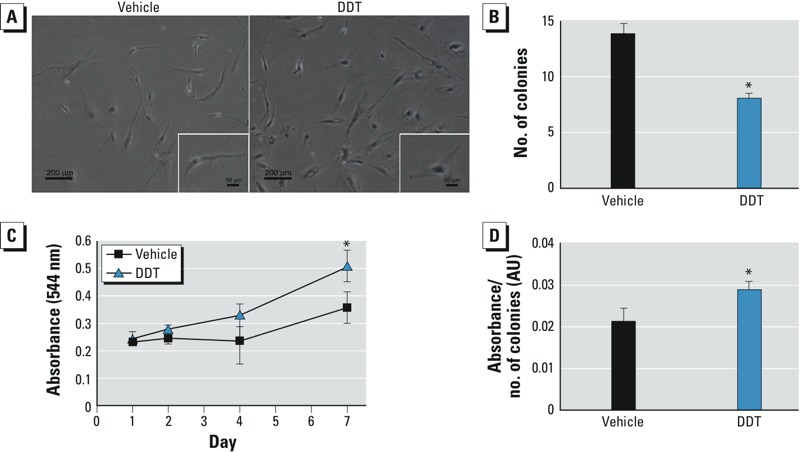
Morphology, self-renewal capacity, and proliferation in MSCs treated with DMSO vehicle or 1 μM DDT for 5 days. (*A*) Representative images of treated MSCs; bars represent 200 μm (4× magnification) and 50 μm (insets; 20× magnification). (*B*) Treated cells were plated at low density, incubated for 14 days, and fixed and stained with crystal violet. Only colonies > 2 mm^2^ in diameter were counted. (*C*) Treated cells were seeded in a 96-well plate and analyzed on days 1, 2, 4, and 7 by the MTT assay. (*D*) Colonies stained with crystal violet were destained, and absorbance was measured on a plate reader at 584 nm; values represent the ratio of absorbance to the number of CFUs. For details of experiments, see “Materials and Methods.” Values (mean ± SD) represent triplicates and three independent experiments for each of the three donors (*n* = 27). *p < 0.05, compared with vehicle treatment alone.

*DDT altered morphology of MSCs and inhibited self-renewal capacity*. To examine the effect of DDT on MSC morphology, we treated cells with DDT in CCM for 5 days. DDT-treated MSCs displayed less fibroblast-like phenotype and more spherical morphology under light microscopy (Figure 1A).

To assess self-renewal capacity, we plated vehicle- and DDT-treated MSCs at low densities and counted colonies per plate after 14 days. The DDT-treated MSCs formed 8.1 ± 0.77 CFUs/plate compared with 13.9 ± 1.56 CFUs/plate in the control group, indicating that DDT reduced the self-renewal of MSCs (Figure 1B). The colonies generated by DDT-treated cells were larger than those from vehicle-treated MSCs, suggesting that the MSCs that were able to self-renew also had greater proliferation potential. As analyzed by an MTT assay, MSCs exposed to DDT demonstrated increased proliferation by day 7 compared with control MSCs (Figure 1C). For quantitation of the CFUs formed by MSCs, crystal violet was extracted and normalized to the number of CFUs in each sample. Vehicle- and DDT-treated MSCs were 0.021 ± 0.0032 AU and 0.029 ± 0.0021 AU, respectively (*p* < 0.05), indicating that the DDT-treated MSCs produced larger CFUs (Figure 1D). These results indicate that DDT reduced the self-renewal of MSCs but that MSCs that were able to self-renew had enhanced proliferative capabilities.

**Figure 2:**
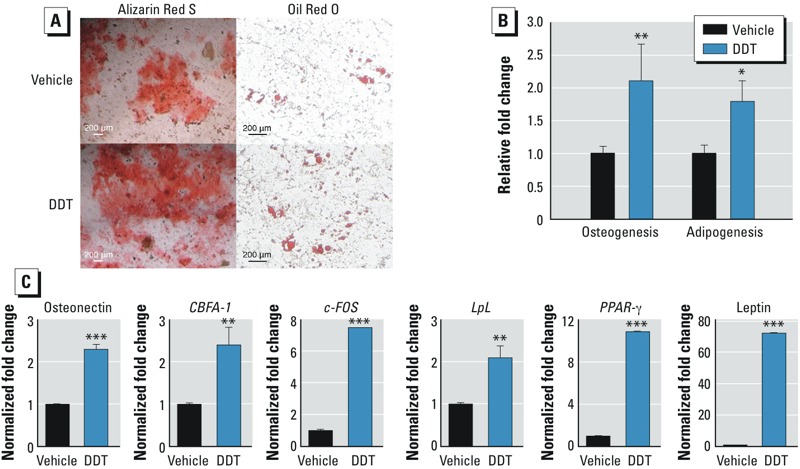
Differentiation and transcription factor expression of MSCs cultured in CCM and treated with DMSO vehicle or 1 μM DDT for 5 days. (*A*) Representative images showing osteogenic (left) and adipogenic differentiation (right); Bars = 200 μm (4× magnification). (*B*) Quantification of differentiation. Stains were eluted and absorbance values were obtained on a plate reader at 584 nm; samples normalized to the amount of protein present. (*C*) qRT-PCR analysis of total cellular RNA prepared from vehicle- and DDT-treated cells; values were normalized to β‑*actin*. For details of experiments, see “Materials and Methods.” Values (mean ± SD) represent triplicates and three independent experiments for each of the three donors (*n* = 27). *p < 0.05, **p < 0.01, and ***p < 0.001, compared with vehicle treatment alone.

*DDT enhanced the differentiation of MSCs*. The differentiation potential of MSCs was evaluated by culturing cells in high-density cultures in either osteogenic or adipogenic induction media. To determine the optimal concentration and the period of treatment, cells were treated with vehicle, 100 nM DDT, or 1 μM DDT for 1 or 5 days. MSCs treated with 1 μM DDT for 5 days demonstrated the greatest osteogenic and adipogenic differentiation relative to control MSCs (Figure 2A,B; see also Supplemental Material, Figure S1). Therefore, all additional MSC experiments were performed using 1 μM DDT for 5 days. MSCs exposed to 1 μM DDT demonstrated increased osteogenic and adipogenic differentiation to 2.1-fold and 1.8-fold respectively, compared with vehicle-treated MSCs. Furthermore, analysis of osteogenic transcription factors in DDT-treated MSCs demonstrated increased mRNA expression of osteonectin, *CBFA-1* (core binding factor alpha 1), and *c-Fos*, with values of 2.3-, 2.4-, and 7.5-fold, respectively, compared with controls (*p* < 0.01; Figure 2C). Analysis of the expression of adipogenic transcription factors in DDT-treated MSCs revealed an increase in mRNA expression of leptin, *LpL* (lipoprotein lipase), and *PPAR*γ (peroxisome proliferator-activated receptor gamma), with values of 2.1-, 10.9-, and 72.3-fold, respectively, compared with control MSCs, indicating increased adipogenesis (*p* < 0.01; Figure 2C).

**Figure 3:**
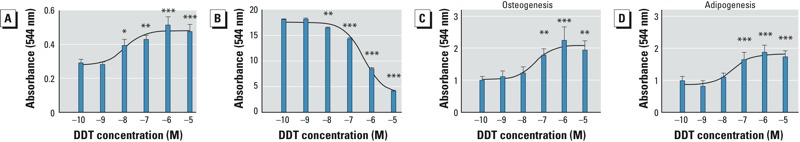
Proliferation, self-renewal, and differentiation of MSCs cultured with 10^–10^ M, 10^–9^ M, 10^–8^ M, 10^–7^ M, 10^–6^ M, or 10^–5^ M DDT for 5 days. (*A*) Proliferation determined using the MTT assay; treated cells were plated (500 cells/well) in a 96-well plate and analyzed 7 days later. (*B*) Self-renewal of treated cells that were plated at low density for 14 days, and fixed and stained with crystal violet; only colonies > 2 mm^2^ in diameter were counted. (*C,D*) Quantification of osteogenic (*C*) or adipogenic (*D*) differentiation. All samples were normalized to the amount of protein present. For details of experiments, see “Materials and Methods.” Values (mean ± SD) represent triplicates and three independent experiments for each of the three donors (*n* = 27). *p < 0.05, **p < 0.01, and ***p < 0.001, compared with vehicle treatment alone.

*DDT impaired innate stem cell properties.* We assessed the DDT concentrations that impair MSC function by treating cells (*n* = 3 donors) with different concentrations of DDT for 5 days and then plating cells for proliferation, CFUs, and differentiation assays. DDT acted on MSC proliferation, CFU formation, and differentiation in a dose-dependent manner. Higher concentrations of DDT resulted in increased proliferation, reduced CFU, and enhanced osteogenic and adipogenic differentiation. The EC_50_ (median effective concentration) of DDT on proliferation was 102.4 nM (Figure 3A). The IC_50_ (median inhibitory concentration) of DDT for self-renewal capacity was 4.2 nM, suggesting that DDT induced robust changes in MSC biology (Figure 3B); the EC_50_ values of DDT for osteogenic and adipogenic differentiation were 382.2 nM and 287.7 nM, respectively (Figure 3C). The results also suggest that the effect of DDT plateaued at 1 μM. These experiments indicate that 1 μM DDT is the optimal dose to elicit a significant response in proliferation, differentiation, and self-renewal of MSCs.

**Figure 4:**
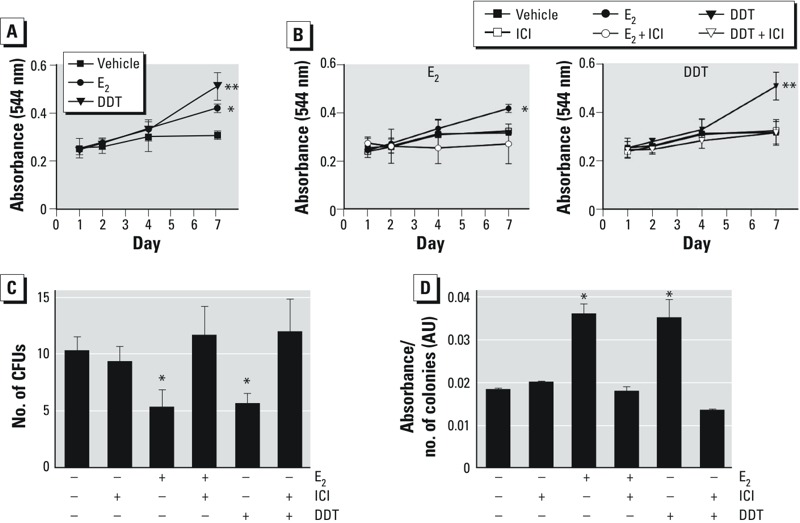
Effects of the estrogen inhibitor ICI on proliferation and self-renewal capacity of MSCs. MSCs cultured in CDS‑FBS with DMSO vehicle, 100 nM ICI, 10 nM E_2_, 10 nM E_2_ + 100 nM ICI, 1 μM DDT, or 1 μM DDT + 100 nM ICI for 5 days. (*A*) Proliferation of MSCs treated with vehicle, E_2_, or DDT was assessed on days 1, 2, 4, and 7 by the MTT assay. (*B*) Proliferation of MSCs treated with vehicle, ICI, E_2_, E_2_ + ICI, DDT, or DDT + ICI was assessed on day 7. (*C*) Self-renewal capacity of MSCs; treated MSCs were plated in triplicate at low density (1.8 cells/cm^2^), cultured for 14 days, and stained with crystal violet. Only colonies > 2 mm^2^ in diameter were counted. (*D*) Colony formation was quantified by eluting crystal violet from CFUs and measuring absorbance on a plate reader at 584 nm; values represent the ratio of absorbance to the number of CFUs. For details of experiments, see “Materials and Methods.” Values (mean ± SD) represent triplicates and three independent experiments for each of the three donors (*n* = 27). *p < 0.05, and **p < 0.01, compared with vehicle treatment alone.

*The ER antagonist ICI inhibited the effects of estrogen and DDT*. We assessed the estrogen-like activity of DDT on MSCs by culturing cells in CDS-FBS and treating them with ICI, E_2_, DDT, or vehicle for 5 days. MSCs were collected on days 1, 2, 4, and 7 and quantified for proliferation. Both E_2_- and DDT-treated MSCs demonstrated enhanced proliferation compared with vehicle-treated MSCs (*p* < 0.05; Figure 4A).

To inhibit estrogenic activity, we assessed cell proliferation in MSCs treated with vehicle, E_2_, or DDT with or without ICI. The enhanced MSC proliferation induced by E_2_ or DDT was blocked by ICI (Figure 4B), indicating that the proliferation of MSCs is mediated by the ER.

For the assessment of self-renewal potential, MSCs were plated at low densities in CDS-FBS containing vehicle, E_2_, or DDT with or without ICI. After 14 days, the MSCs were stained with crystal violet for quantification of CFUs. The MSCs treated with E_2_ or DDT showed diminished self-renewal capacity, forming 5.3 and 5.6 colonies, respectively (*p* < 0.05). Again, ICI treatment inhibited the effect of E_2_ or DDT on MSC self-renewal (Figure 4C).

Because CFUs formed with MSCs cultured in FBS and treated with DDT were 1.4 times larger in size than vehicle-treated MSCs, we analyzed cells cultured in CDS-FBS and treated with E_2_ or DDT. CCM was switched to CDS-FBS media to examine the effects of E_2_ and DDT, without interference from endogenous E_2_ in the serum. Absorbance values for MSCs treated with E_2_ and DDT were 0.036 ± 0.0025 AU and 0.035 ± 0.0044 AU, respectively (*p* < 0.05, compared with vehicle-treated cells (Figure 4D). Thus, the colony sizes of E_2_- or DDT-treated MSCs were 2.1 times that of vehicle-treated cells. In MSCs treated with E_2_ + ICI (0.018 ± 0.0012 AU) or DDT + ICI (0.015 ± 0.0003 AU), colony size was equivalent to that of vehicle-treated MSCs, suggesting that ER inhibitors returned the colony size to baseline (Figure 4D).

**Figure 5:**
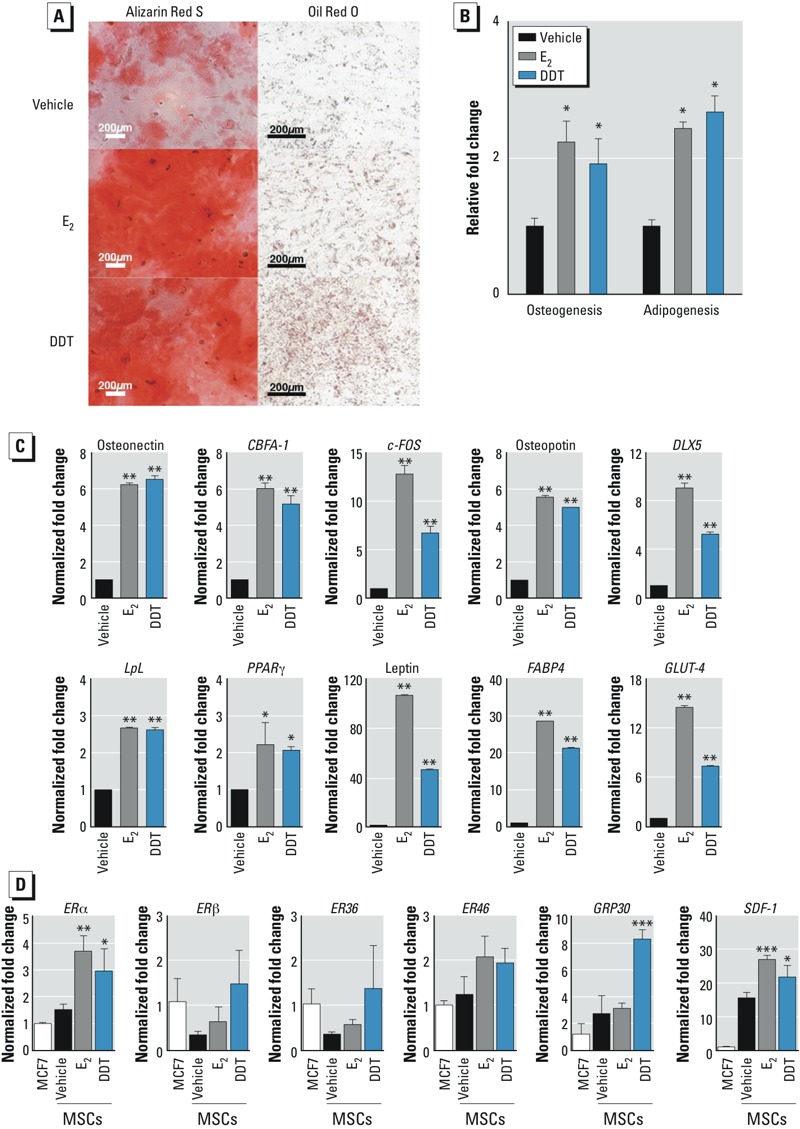
Effects of E_2_ and DDT on osteogenic and adipogenic differentiation of MSCs. (*A*) MSCs were cultured in CDS‑FBS with vehicle, 10 nM E_2_, or 1 μM DDT for 5 days. The medium was changed to osteogenic or adipogenic differentiation medium. After 14 days, MSCs were fixed and stained with Alizarin Red S for osteogenic differentiation and Oil Red O for adipogenic differentiation. (*A*) Representative images showing osteogenic (left) and adipogenic differentiation (right); bars = 200 μm (4× magnification for bone differentiation images,and 10× magnification for fat differentiation images). (*B*) Quantification of osteogenic and adipogenic differentiation. Stains were extracted and absorbance was measured; samples were normalized to the amount of protein present. (*C*) qRT-PCR analysis of total cellular RNA prepared from cells cultured in CDS‑FBS and treated with vehicle, E_2_, or DDT; Values are presented as fold change relative to vehicle-treated MSCs. (*D*) qRT-PCR analysis of total cellular RNA prepared from MCF7 cells (positive control) or MSCs treated with vehicle, E_2_, or DDT was analyzed. Values (mean ± SD) represent triplicates and three independent experiments for each of the three donors (*n* = 27). *p < 0.05, **p < 0.01, and ***p < 0.001, compared with vehicle treatment alone.

*DDT mediated MSC differentiation via an E_2_-dependent mechanism*. The estrogenic effects of DDT on MSCs were examined by culturing cells in CDS-FBS, followed by treatment with E_2_, DDT, or vehicle for 5 days. MSCs were then switched to either osteogenic or adipogenic induction media. After 14 days, MSCs were assessed for osteogenic and adipogenic differentiation. MSCs treated with E_2_, DDT, or vehicle all demonstrated differentiation along osteogenic and adipogenic lineages (Figure 5A). Quantification of E_2_-treated MSCs demonstrated enhanced osteogenic and adipogenic differentiation to 2.2- and 2.4-fold, respectively, compared with vehicle-treated MSCs; for MSCs treated with DDT, differentiation was enhanced to 1.9- and 2.7-fold, respectively (Figure 5B).

To assess the influence of E_2_ and DDT on osteogenic and adipogenic factors, we analyzed treated cells by qRT-PCR. The expression levels of key factors involved in osteogenic and adipogenic differentiation increased after exposure to E_2_ or DDT (*p* < 0.05; Figure 5C). The mRNA expression of osteogenic factors increased in E_2_-treated and DDT-treated MSCs relative to vehicle after treatment, with mRNA levels as follows: osteonectin, 6.2- and 6.5-fold for E_2_ and DDT, respectively; *CBFA-1*, 6.0- and 5.2-fold; *c-Fos*, 12.7- and 6.3-fold; osteopontin, 5.6- and 5.0-fold; and *DLX5,* 9.0- and 5.2-fold (*p* < 0.05; Figure 5C). Similarly, the expression of adipogenic factors was increased after treatment: *LpL*, 2.7- and 2.6-fold after treatment with E_2_ and DDT, respectively; *PPAR*γ, 2.2- and 2.1-fold; leptin, 106.7- and 46.4-fold; *FABP4,* 28.6- and 21.3-fold; and *GLUT-4*, 14.5- and 7.3-fold (*p* < 0.05; Figure 5C).

We conducted additional analysis to determine whether MSCs express ERα, ERβ, and/or ER variants, including ER36, ER46, and GRP30, and whether E_2_ or DDT simulated the expression of these receptors. We used MCF7 cells, an ER-positive breast cancer cell line, as a positive control for the detection of ER and ER variants. Vehicle-treated MSCs expressed comparable levels of ERα, ER46, and GRP30. The addition of E_2_ enhanced the expression of ERα and ER46 in MSCs, but DDT enhanced the expression of ERα and GRP30 (Figure 5D; *p* < 0.05). To assess the function of ERs, we monitored the mRNA levels of *SDF-1* because SDF-1 is a downstream target of ER. Increased mRNA expression levels of *SDF-1* were observed in MSCs treated with E_2_- or DDT (Figure 5E; *p* < 0.05).

**Figure 6:**
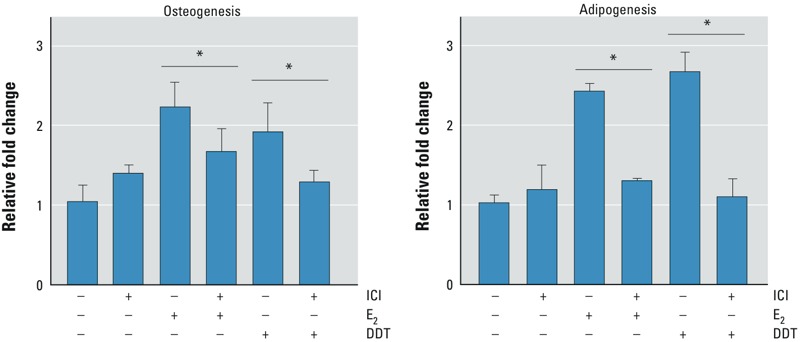
Effects of ER inhibition by ICI on osteogenic (left) and adipogenic (right) differentiation of MSCs treated with vehicle, E_2_, or DDT. MSCs were treated with DMSO vehicle, 100 nM ICI, 10 nM E_2_, 10 nM E_2_ + 100 nM ICI, 1 μM DDT, or 1 μM DDT + 100 nM ICI for 5 days. The medium was changed to CDS‑FBS containing osteogenic or adipogenic differentiation supplements and incubated for 14 days. MSCs were fixed and stained with Alizarin Red S for osteogenic differentiation or Oil Red O for adipogenic differentiation; to quantify the amount of differentiation, stains were eluted from the plates and read on a plate reader at 584 nm. Samples were normalized to the amount of protein present. Values (mean ± SD) represent triplicates and three independent experiments for each of the three donors (*n* = 27). *p < 0.05.

In MSCs treated with ICI in the presence of E_2_ or DDT, both osteogenic and adipogenic differentiation of MSCs were reduced, suggesting that the enhanced differentiation of DDT-treated MSCs is, in part, through the activation of ER signaling (Figure 6).

*RNA-seq analysis reveals distinct gene expression profiles for DDT-treated MSCs*. To globally investigate the influences of DDT on MSC signaling pathways, we examined the gene expression profiles of MSCs treated with either DDT or vehicle using RNA-seq. A total of 121 genes and noncoding RNAs were differentially expressed at statistically significant levels (see Supplemental Material, Figure S2, Tables S3,S4). Gene clustering revealed two separate clusters, and Ingenuity Pathway Analysis software found the two most significantly affected pathways were “cell death and survival, tumor morphology, cancer” and “RNA posttranscriptional modification, cellular assembly and organization, cellular development” (see Supplemental Material, Figure S3, Table S3).

## Discussion

To our knowledge, this is one of the first studies to examine the effects of DDT on human MSCs. The results show increased proliferation, diminished self-renewal capacity, and enhanced differentiation in DDT-treated MSCs. We also observed increased expression of mRNAs for several key factors that regulate either adipogenesis or osteogenesis. In MSCs treated with DDT plus ICI, pluripotency, proliferation, and self-renewal potential were equivalent to that of vehicle-treated cells, indicating that DDT functions through ER signaling pathways.

Previous studies have focused on the impact of DDT on the reproductive system. However, the results of the present study suggest that DDT has negative effects on adult stem cells. Because MSCs regulate hematopoietic stem cell development and maturation, alterations in MSC biology may disrupt bone marrow homeostasis. Furthermore, alterations in MSCs have been linked to metabolic diseases and obesity. Because MSCs can also give rise to adipocytes, it is of particular interest to determine whether exposure to DDT can directly modulate lipogenesis, lipolysis, and adipogenesis. It will be important to understand how the set point for adipocyte number is programmed in adults and how alterations by DDT exposure may promote obesity in adults.

Consistent with previous studies investigating the biological action of DDT in reproductive cells and cancer cells, our results clearly demonstrate an E_2_-like response of DDT in adult MSCs. E_2_ has been shown to influence MSCs by enhancing the proliferative capacity and attenuating the self-renewal capacity (Di Gregorio et al. 2001; Hong et al. 2011), and to diminish self-renewal capacity through the activation of ERα (Di Gregorio et al. 2001). Furthermore, E_2_ enhances osteogenic differentiation through ERα and ERβ, and adipogenesis via the ERα receptor only (Hong et al. 2006; Rajalin et al. 2010). Results of the present study suggest that ERα and ERβ are expressed by adipose stem cells; however, ERα was significantly simulated by E_2_ and DDT. Furthermore, DDT may signal through the ER variant GRP30 because its expression was significanly enhanced following stimulation by DDT. It should also be noted that although DDT reduced self-renewal capacity, those MSCs that retained the potential to self-renew demonstrated enhanced proliferation. Together, these results suggest that DDT enriched a subpopulation of MSCs with the potential to self-renew and proliferate rapidly. Our data corroborate that DDT treatment can result in changes in MSCs that are similar to those induced by E_2_. Furthermore, the use of ER inhibitors markedly reduced the effects of DDT, providing further support of the activation of DDT through ER signaling.

The global assessment of the gene expression profile of MSCs exposed to DDT demonstrated altered expression of genes involved with cell death and survival, cancer, and cellular assembly and organization. The results from this study provide support for previous population and molecular studies that have indicated increased cancer incidence in children and adults exposed to environmental DDT (Bratton et al. 2012; Cohn et al. 2007; Diel et al. 2002; Guo et al. 2013; Kazantseva et al. 2013). Our data indicate that DDT altered MSC biology and suggest that these changes could result in higher cancer incidence in individuals subjected to long-term exposure. Previous studies have reported that alterations in adult stem cells contributed to the enhancement of tumorigenicity (Strong et al. 2012, 2013b); results of those studies also provide support to future studies investigating the interplay between EDCs, adult stem cells, and cancer incidence.

Numerous studies have demonstrated that DDT and other chlorinated biphenyl pesticides exert estrogenic activity at both the cellular and molecular levels (Bratton et al. 2012; Diel et al. 2002; Li et al. 2013), but few, if any, have studied the biological changes induced by these chemicals in model systems capable of assessing differentiation outcomes and cell fate, such as human MSCs. The use of adult human MSCs provides a powerful biological system to interrogate the molecular mechanisms underlying the effects of EDCs. In particular, MSCs can be treated with different EDCs and analyzed for self-renewal capacity, proliferative rate, and differentiation into several lineages. The data presented here describe the altered gene expression profile of MSCs after 5 days of exposure to DDT, but additional research is required to determine the impact of long-term DDT exposure on MSC signaling pathways, differentiation, and functionality. Impairment in any aspect of self-renewal or differentiation of the MSCs after treatment with EDCs indicates a significant alteration in the biology of these stem cells and suggests a potential cause of increased cancer incidence due to long-term exposure.

## Supplemental Material

(1.5 MB) PDF
