# Supplementary material for: Effects of the Endocrine-Disrupting Chemical DDT on Self-Renewal and Differentiation of Human Mesenchymal Stem Cells
Source: Environ Health Perspect. 2014 Jul 11;123(1):42–8. doi: 10.1289/ehp.1408188 (PMC4286277; doi:10.1289/ehp.1408188)
Supplement: (1.5 MB) PDF [file ehp.1408188.s001.508.pdf]

Environmental Health Perspectives doi:10.1289/ehp.1408188

Supplemental Material

Effects of the Endocrine-Disrupting Chemical DDT on Self-Renewal and Differentiation of Human Mesenchymal Stem Cells

Amy L. Strong, Zhenzhen Shi, Michael J. Strong, David F.B. Miller, Douglas B. Rusch, Aaron M. Buechlein, Erik K. Flemington, John A. McLachlan, Kenneth P. Nephew, Matthew E. Burow, and Bruce A. Bunnell

Table of Contents **Page**

Figure S1. MSCs exposed to 5 day treatment of high DDT concentration demonstrated the greatest increase in osteogenesis and adipogenesis 2

Figure S2. Cluster analysis of DDT-treated MSCs relative to vehicle-treated MSCs (DMSO) 3

Figure S3. DDT treated MSCs display altered gene expression profiles that can be linked into two distinct canonical pathways 5

Table S1. Donors' demographic information 6

Table S2. Primer sequences 7

Table S3. DDT-treated MSCs overexpress genes involved in cell death and survival and RNA post-transcriptional modification. 8

Table S4. Differentially expressed genes in DDT-treated MSCs compared to vehicle-treated MSCs 10

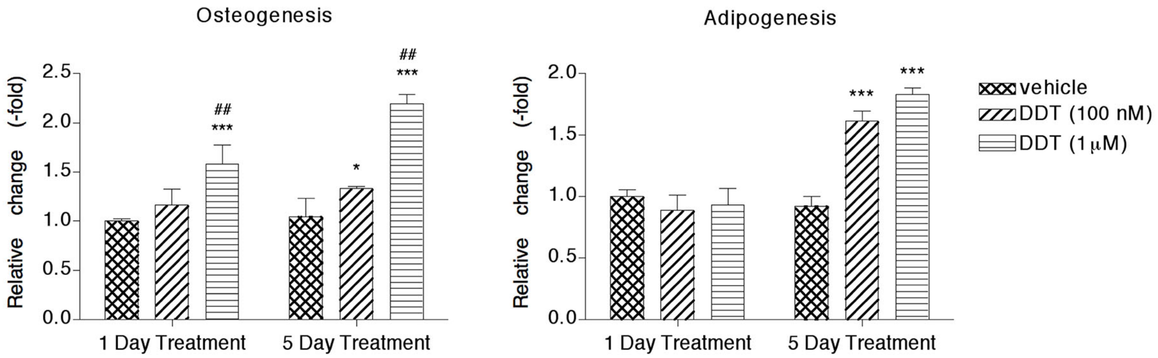

Figure S1. MSCs exposed to 5 day treatment of high DDT concentration demonstrated the greatest increase in osteogenesis and adipogenesis. MSCs were cultured in CCM and treated with vehicle only (DMSO), 100 nM DDT or 1 μM DDT for 1 or 5 days. (A) After the treatment period, the medium was switched to osteogenic or adipogenic differentiation medium. After 14 days, MSCs were fixed and stained with Alizarin Red S for osteogenic differentiation and Oil Red O for adipogenic differentiation. To quantify the differentiation after treatment with DDT, cells stained with Alizarin Red S were eluted with 10% CPC, and cells stained with Oil Red O were destained with isopropanol. Absorbance values were obtained on a plate reader at 584 nm. Protein extraction with RIPA buffer and protein quantification with the BCA assay was used to normalize the amount of protein in each sample. Changes in osteogenic and adipogenic differentiation after DDT treatment were compared to 1 day treatment of MSCs vehicle. Bars, ± SD. *, $P < 0.05$, ***$P < 0.001$ compared to vehicle-treated MSCs, ##, $P < 0.01$ compared to 100 nM DDT treated MSCs.

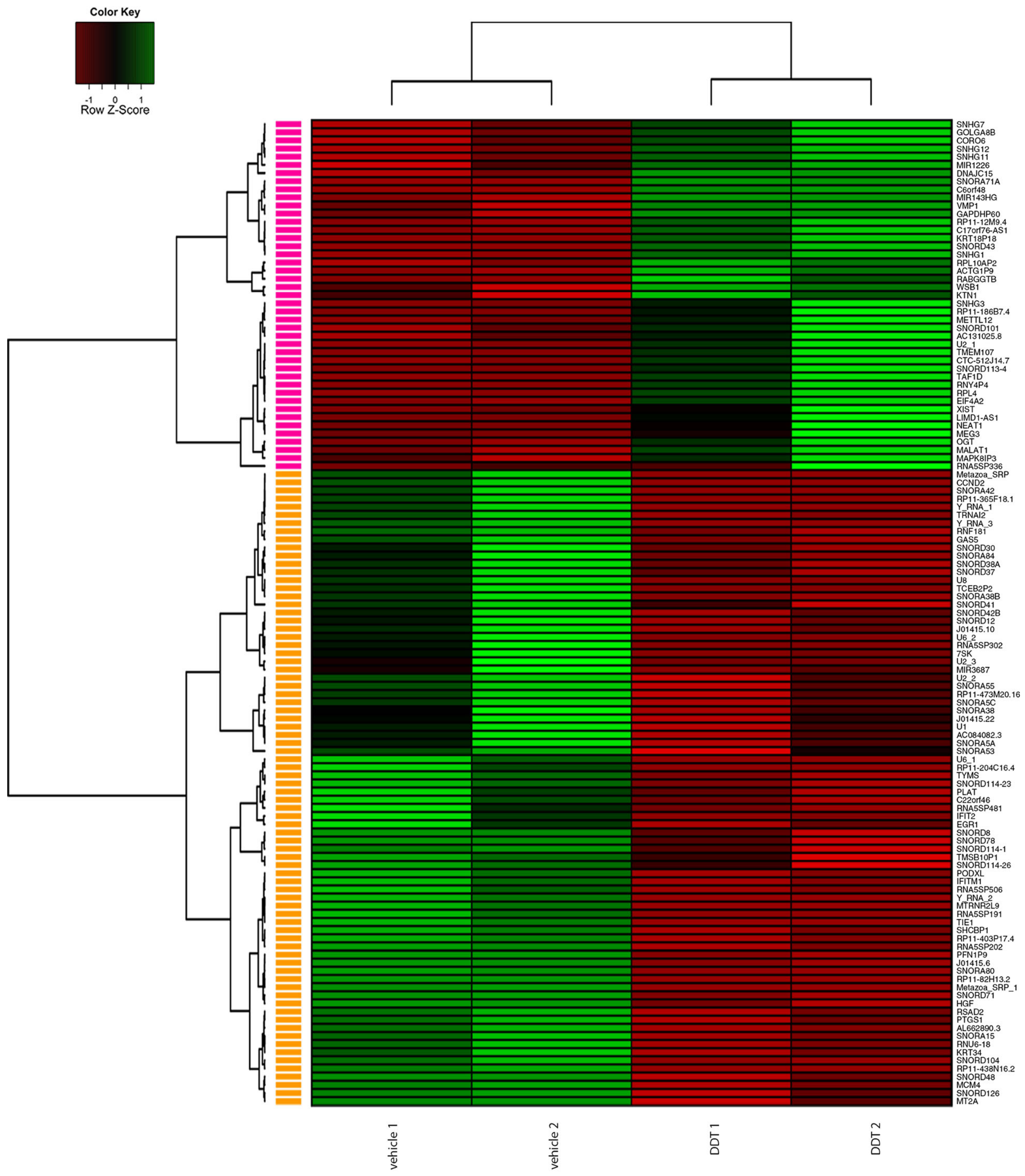

Figure S2. Cluster analysis of DDT-treated MSCs relative to vehicle-treated MSCs (DMSO). Technical replicates of vehicle-treated MSCs and DDT-treated MSCs from the same donor were sequenced and grouped using hierarchical clustering. Differentially expressed genes and non-coding RNAs between the samples are displayed using an expression heat map. EdgeR was used

in the differential gene expression analysis. *Z*-scores represent the expression of each gene normalized to the mean across all four samples. Statistical significance was determined by an adjusted P value < 0.05.

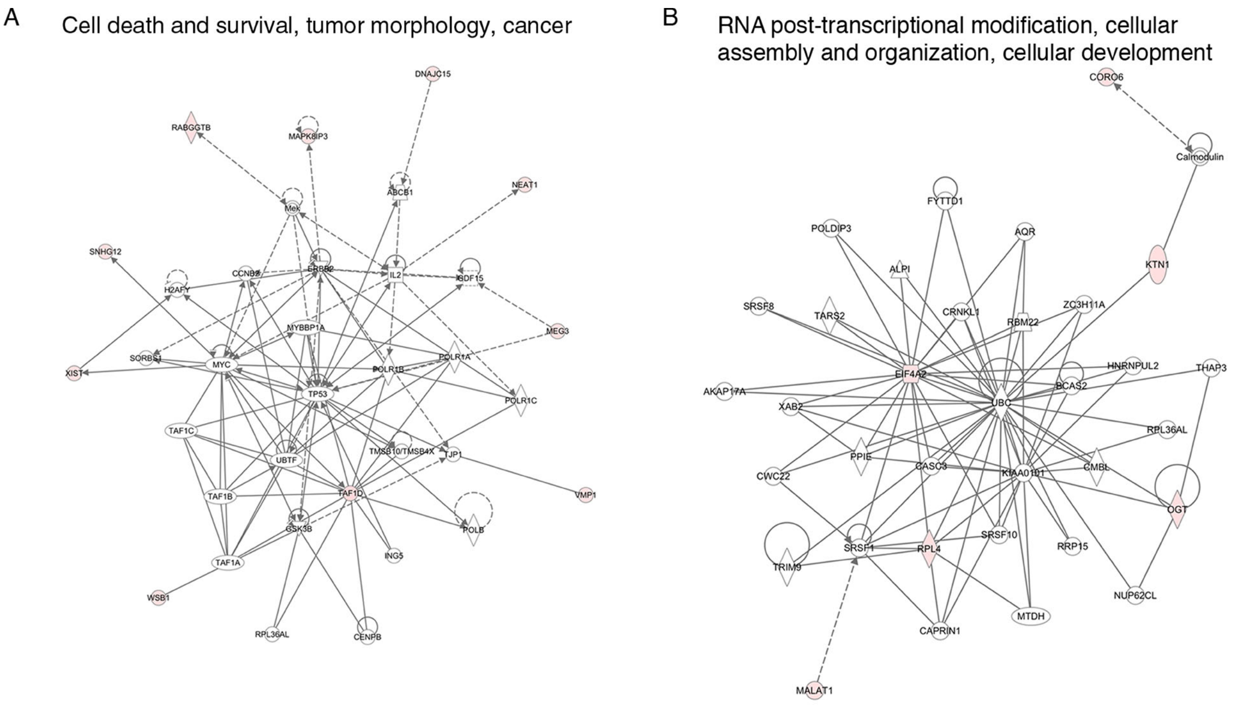

Figure S3. DDT treated MSCs display altered gene expression profiles that can be linked into two distinct canonical pathways. Differentially expressed genes identified with RNA-seq were analyzed using IPA. Two distinct canonical pathways were identified: (A) cell death and survival, tumor morphology, cancer and (B) RNA post-transcriptional modification, cellular assembly and organization, cellular development.

Table S1. Donors' demographic information.

Donor	Race	Sex	Age	BMI
1	Caucasian	Female	22	21.8
2	Caucasian	Female	33	19.4
3	Caucasian	Female	21	22.7

Table S2. Primer sequences.

Gene	Forward	Reverse
ON	5’-TGTGGGAGCTAATCCTGTCC-3’	5’-TCAGGACGTTCTTGAGCCAGT-3’
CBFA-1	5’-CTCACTACCACACCTACCTG-3’	5’-TCAATATGGTCGCCAAACAGATTC-3’
c-FOS	5’-CCTGTCAAGAGCATCAGCAG-3’	5’-GTCAGAGGAAGGCTCATTGC-3’
OPN	5’-GCTCTAGAATGAGAATTGCACTG-3’	5’-TGTCGGTCCTGAGGTAACTG-3’
DLX5	5’-TGGCCCGAGTCTTCAGCTAC’-3’	5’-TGGTTGGTCGGTCTCTTTCT-3’
LPL	5’-GAGATTTCTCTGTATGGCACC-3’	5’-CTGCAAATGAGACACTTTCTC-3’
PPAR-Y	5’-GCTGTTATGGGTGAAACTCTG-3’	5’-ATAAGGTGGAGATGCAGGTTC-3’
LEP	5’-GGCTTTGGCCCTATCTTTTC-3’	5’-GCTCTTAGAGAAGGCCAGCA-3’
FABP4	5’-CTCTAGAATGAGAATTGCACTG-3’	5’-TAGTTTAAGGACCGGGTCAT-3’
GLUT4	5’-AGCAGCTCTCTGGCATCAAT-3’	5’-CAATGGAGACGTAGCACATG-3’
β-actin	5’-CACCTTCTACAATGAGCTGC-3’	5’-TCTTCTCGATGCTCGACGGA-3’
ERα	5’-GGCATGGTGGAGATCTTCGA-3’	5’-CCTCTCCCTGCAGATTCATCA-3’
ERβ	5’-CGTGACCGATGCTTTGGTTT-3’	5’-CGTGACCGATGCTTTGGTTT-3’
ER46	5’-CCAGGGTGGCAGAGAAAG –3’	5’-CTCTCAGACTGAGGCAGGGAAACC-3’
ER36	5’-CAAGTGGTTTCCTCGTGTCTAAAGC-3’	5’-TGTTGAGTGTTGGTTCCAGG-3’
GRP30	5’-GGCAGGTACCCAGAGAGTGA-3’	5’-CTTGAAGTGAGCCTGGCATT-3’
SDF-1	5’-ACACTCCAAACTGTGCCCTTCA-3’	5’-CCACGTCTTTGCCCTTTCATC-3’

Table S3. DDT-treated MSCs overexpress genes involved in cell death and survival and RNA post-transcriptional modification. MSCs were treated with DDT (1 μM) or vehicle for five days in complete culture media replaced every 2-3 days. Values are represented as DDT-treated MSCs relative to vehicle-treated MSCs. All values are statistically significant ($P < 0.05$).

Pathway	Ensembl	Symbol	Entrez gene name	Fold change	Location
Cell Death and Survival, Tumor Morphology, Cancer	ENSG00000166012	*TAF1D*	TATA box binding protein (TBP)-associated factor	7.29	Nucleus
Cell Death and Survival, Tumor Morphology, Cancer	ENSG00000197989	*SNHG12*	small nucleolar RNA host gene 12	3.75	unknown
Cell Death and Survival, Tumor Morphology, Cancer	ENSG00000137955	*RABGGTB*	Rab geranylgeranyltransferase, beta subunit	3.52	Cytoplasm
Cell Death and Survival, Tumor Morphology, Cancer	ENSG00000214548	*MEG3*	maternally expressed 3	2.45	unknown
Cell Death and Survival, Tumor Morphology, Cancer	ENSG00000229807	*XIST*	X inactive specific transcript	2.23	Nucleus
Cell Death and Survival, Tumor Morphology, Cancer	ENSG00000138834	*MAPK8IP3*	mitogen-activated protein kinase 8 interacting protein 3	2.18	Cytoplasm
Cell Death and Survival, Tumor Morphology, Cancer	ENSG00000120675	*DNAJC15*	DnaJ (Hsp40) homolog, subfamily C, member 15	2.12	Cytoplasm
Cell Death and Survival, Tumor Morphology, Cancer	ENSG00000245532	*NEAT1*	nuclear paraspeckle assembly transcript 1	1.96	unknown
Cell Death and Survival, Tumor Morphology, Cancer	ENSG00000109046	*WSB1*	WD repeat and SOCS box containing 1	1.84	unknown
Cell Death and Survival, Tumor Morphology, Cancer	ENSG00000062716	*VMP1*	vacuole membrane protein 1	1.63	Plasma membrane
RNA Post-Transcriptional Modification, Cellular Assembly and Organization, Cellular Development	ENSG00000251562	*MALAT1*	metastasis associated lung adenocarcinoma transcript 1	1.71	Nucleus
RNA Post-Transcriptional Modification, Cellular Assembly and Organization, Cellular Development	ENSG00000147162	*OGT*	O-linked N-acetylglucosamine (GlcNAc) transferase	1.73	Cytoplasm
RNA Post-Transcriptional Modification, Cellular Assembly and Organization, Cellular Development	ENSG00000126777	*KTN1*	kinectin 1 (kinesin receptor)	1.74	Cytoplasm

Pathway	Ensembl	Symbol	Entrez gene name	Fold change	Location
RNA Post-Transcriptional Modification, Cellular Assembly and Organization, Cellular Development	ENSG00000156976	*EIF4A2*	eukaryotic translation initiation factor 4A2	2.39	Cytoplasm
RNA Post-Transcriptional Modification, Cellular Assembly and Organization, Cellular Development	ENSG00000167549	*CORO6*	coronin 6	3.74	Extracellular Space
RNA Post-Transcriptional Modification, Cellular Assembly and Organization, Cellular Development	ENSG00000174444	*RPL4*	ribosomal protein L4	3.93	Cytoplasm
RNA Post-Transcriptional Modification, Cellular Assembly and Organization, Cellular Development	ENSG00000147162	*OGT*	O-linked N-acetylglucosamine (GlcNAc) transferase	1.73	Cytoplasm
RNA Post-Transcriptional Modification, Cellular Assembly and Organization, Cellular Development	ENSG00000126777	*KTN1*	kinectin 1 (kinesin receptor)	1.74	Cytoplasm
RNA Post-Transcriptional Modification, Cellular Assembly and Organization, Cellular Development	ENSG00000156976	*EIF4A2*	eukaryotic translation initiation factor 4A2	2.39	Cytoplasm
RNA Post-Transcriptional Modification, Cellular Assembly and Organization, Cellular Development	ENSG00000167549	*CORO6*	coronin 6	3.74	Extracellular Space
RNA Post-Transcriptional Modification, Cellular Assembly and Organization, Cellular Development	ENSG00000174444	*RPL4*	ribosomal protein L4	3.93	Cytoplasm

Table S4. Differentially expressed genes in DDT-treated MSCs compared to vehicle-treated MSCs.

Ensembl	Symbol	Entrez gene name	Fold change	Location	Function
ENSG00000263764	*SNORD43*	small nucleolar RNA, C/D box 43	47414.987	unknown	other
ENSG00000225091	*SNORA71A*	small nucleolar RNA, H/ACA box 71A	672.849	unknown	other
ENSG00000201672	*SNORD113-4*	small nucleolar RNA, C/D box 113-4	672.849	unknown	other
ENSG00000261764	N/A	unidentified	65.868	N/A	N/A
ENSG00000179029	*TMEM107*	transmembrane protein 107	42.749	unknown	other
ENSG00000230530	*LIMD1-AS1*	LIMD1 antisense RNA 1	24.694	unknown	other
ENSG00000264772	*SNORA67*	small nucleolar RNA, H/ACA box 67	20.728	unknown	other
ENSG00000215252	*GOLGA8A/ GOLGA8B*	golgin A8 family, member B	15.689	Cytoplasm	other
ENSG00000255717	*SNHG1*	small nucleolar RNA host gene 1 (non-protein coding)	14.628	unknown	other
ENSG00000213857	N/A	unidentified	10.834	N/A	N/A
ENSG00000166012	*TAF1D*	TATA box binding protein (TBP)-associated factor, RNA polymerase I, D, 41kDa	7.291	Nucleus	other
ENSG00000242125	*SNHG3*	small nucleolar RNA host gene 3 (non-protein coding)	7.201	Nucleus	other
ENSG00000214756	*METTL12*	methyltransferase like 12	6.793	unknown	other
ENSG00000221585	*mir-1226*	microRNA 1226	5.912	Cytoplasm	microRNA
ENSG00000200843	N/A	unidentified	5.279	N/A	N/A
ENSG00000174365	*SNHG11*	small nucleolar RNA host gene 11 (non-protein coding)	5.101	unknown	other
ENSG00000223247	N/A	unidentified	4.735	N/A	N/A
ENSG00000174444	*RPL4*	ribosomal protein L4	3.930	Cytoplasm	enzyme
ENSG00000197989	*SNHG12*	small nucleolar RNA host gene 12 (non-protein coding)	3.750	unknown	other
ENSG00000167549	*CORO6*	coronin 6	3.737	Extracellular Space	other
ENSG00000184188	N/A	unidentified	3.613	N/A	N/A
ENSG00000137955	*RABGGTB*	Rab geranylgeranyltransferase, beta subunit	3.524	Cytoplasm	enzyme
ENSG00000188873	N/A	unidentified	3.406	N/A	N/A
ENSG00000204387	*C6orf48*	chromosome 6 open reading frame 48	2.981	unknown	other
ENSG00000206754	*SNORD101*	small nucleolar RNA, C/D box 101	2.890	unknown	other
ENSG00000201059	*RNA5SP336*	RNA, 5S ribosomal pseudogene 336	2.755	unknown	other
ENSG00000175061	*C17orf76-AS1*	C17orf76 antisense RNA 1	2.634	unknown	other

Ensembl	Symbol	Entrez gene name	Fold change	Location	Function
ENSG00000214548	*MEG3*	maternally expressed 3 (non-protein coding)	2.453	unknown	other
ENSG00000156976	*EIF4A2*	eukaryotic translation initiation factor 4A2	2.389	Cytoplasm	translation regulator
ENSG00000229807	*XIST*	X inactive specific transcript (non-protein coding)	2.228	Nucleus	other
ENSG00000138834	*MAPK8IP3*	mitogen-activated protein kinase 8 interacting protein 3	2.182	Cytoplasm	other
ENSG00000120675	*DNAJC15*	DnaJ (Hsp40) homolog, subfamily C, member 15	2.122	Cytoplasm	other
ENSG00000249669	*MIR143HG*	MIR143 host gene (non-protein coding)	2.036	unknown	other
ENSG00000245532	*NEAT1*	nuclear paraspeckle assembly transcript 1 (non-protein coding)	1.958	unknown	other
ENSG00000248180	N/A	unidentified	1.940	N/A	N/A
ENSG00000233016	*SNHG7*	small nucleolar RNA host gene 7 (non-protein coding)	1.899	unknown	other
ENSG00000229349	N/A	unidentified	1.851	N/A	N/A
ENSG00000109046	*WSB1*	WD repeat and SOCS box containing 1	1.841	unknown	other
ENSG00000253864	N/A	unidentified	1.838	N/A	N/A
ENSG00000126777	*KTN1*	kinectin 1 (kinesin receptor)	1.742	Cytoplasm	transmembrane receptor
ENSG00000147162	*OGT*	O-linked N-acetylglucosamine (GlcNAc) transferase	1.725	Cytoplasm	enzyme
ENSG00000251562	*MALAT1*	metastasis associated lung adenocarcinoma transcript 1 (non-protein coding)	1.713	Nucleus	other
ENSG00000062716	*VMP1*	vacuole membrane protein 1	1.626	Plasma Membrane	other
ENSG00000234741	*GAS5*	growth arrest-specific 5 (non-protein coding)	0.650	unknown	other
ENSG00000210112	N/A	unidentified	0.647	N/A	N/A
ENSG00000199575	*SNORD114-1*	small nucleolar RNA, C/D box 114-1	0.631	unknown	other
ENSG00000200406	*SNORD114-23*	small nucleolar RNA, C/D box 114-23	0.610	unknown	other
ENSG00000200413	*SNORD114-26*	small nucleolar RNA, C/D box 114-26	0.609	unknown	other
ENSG00000212304	*SNORD12*	small nucleolar RNA, C/D box 12	0.597	unknown	other
ENSG00000206775	*SNORD37*	small nucleolar RNA, C/D box 37	0.594	unknown	other
ENSG00000119922	*IFIT2*	interferon-induced protein with tetratricopeptide repeats 2	0.590	Cytoplasm	other
ENSG00000223224	*SNORD71*	small nucleolar RNA, C/D box 71	0.583	unknown	other
ENSG00000207424	*SNORD30*	small nucleolar RNA, C/D box 30	0.569	unknown	other
ENSG00000185885	*IFITM1*	interferon induced transmembrane protein 1	0.566	Plasma Membrane	transmembrane receptor

Ensembl	Symbol	Entrez gene name	Fold change	Location	Function
ENSG00000208317	*SNORD78*	small nucleolar RNA, C/D box 78	0.560	unknown	other
ENSG00000120738	*EGR1*	early growth response 1	0.553	Nucleus	transcription regulator
ENSG00000238423	*SNORD42B*	small nucleolar RNA, C/D box 42B	0.550	unknown	other
ENSG00000238344	*SNORD126*	small nucleolar RNA, C/D box 126	0.547	unknown	other
ENSG00000200785	*SNORD8*	small nucleolar RNA, C/D box 8	0.541	unknown	other
ENSG00000201457	*SNORA55*	small nucleolar RNA, H/ACA box 55	0.539	unknown	other
ENSG00000212443	*SNORA53*	small nucleolar RNA, H/ACA box 53	0.520	unknown	other
ENSG00000209702	*SNORD41*	small nucleolar RNA, C/D box 41	0.519	unknown	other
ENSG00000201823	*SNORD48*	small nucleolar RNA, C/D box 48	0.514	unknown	other
ENSG00000125148	*MT2A*	metallothionein 2A	0.508	Cytoplasm	other
ENSG00000253190	N/A	unidentified	0.504	N/A	N/A
ENSG00000168894	*RNF181*	ring finger protein 181	0.500	unknown	other
ENSG00000200394	*SNORA38B*	small nucleolar RNA, H/ACA box 38B	0.497	unknown	other
ENSG00000228499	N/A	unidentified	0.494	N/A	N/A
ENSG00000223001	N/A	unidentified	0.488	N/A	N/A
ENSG00000202031	*SNORD38A*	small nucleolar RNA, C/D box 38A	0.485	unknown	other
ENSG00000104368	*PLAT*	plasminogen activator, tissue	0.475	Extracellular Space	peptidase
ENSG00000200816	*SNORA38*	small nucleolar RNA, H/ACA box 38	0.473	unknown	other
ENSG00000264063	*MIR3687*	microRNA 3687	0.464	Cytoplasm	microRNA
ENSG00000104738	*MCM4*	minichromosome maintenance complex component 4	0.457	Nucleus	enzyme
ENSG00000206838	*SNORA5A*	small nucleolar RNA, H/ACA box 5A	0.447	unknown	other
ENSG00000239183	*SNORA84*	small nucleolar RNA, H/ACA box 84	0.438	unknown	other
ENSG00000128567	*PODXL*	podocalyxin-like	0.436	Plasma Membrane	kinase
ENSG00000238621	*TRNAI2*	transfer RNA isoleucine 2 (anticodon UAU)	0.417	unknown	other
ENSG00000255262	N/A	unidentified	0.361	N/A	N/A
ENSG00000200792	*SNORA80*	small nucleolar RNA, H/ACA box 80	0.358	unknown	other
ENSG00000201772	*SNORA5C*	small nucleolar RNA, H/ACA box 5C	0.338	unknown	other
ENSG00000201185	*RNA5SP202*	RNA, 5S ribosomal pseudogene 202	0.324	unknown	other
ENSG00000252623	*RNA5SP481*	RNA, 5S ribosomal pseudogene 481	0.319	unknown	other
ENSG00000210196	N/A	unidentified	0.314	N/A	N/A
ENSG00000176890	*TYMS*	thymidylate synthetase	0.307	Nucleus	enzyme
ENSG00000171241	*SHCBP1*	SHC SH2-domain binding protein 1	0.287	unknown	other
ENSG00000238554	N/A	unidentified	0.247	N/A	N/A
ENSG00000131737	*KRT34*	keratin 34	0.237	Cytoplasm	other

Ensembl	Symbol	Entrez gene name	Fold change	Location	Function
ENSG00000261889	N/A	unidentified	0.237	N/A	N/A
ENSG00000207257	N/A	unidentified	0.216	N/A	N/A
ENSG00000201686	*RNA5SP506*	RNA, 5S ribosomal pseudogene 506	0.198	unknown	other
ENSG00000184208	*C22orf46*	chromosome 22 open reading frame 46	0.189	unknown	other
ENSG00000095303	*PTGS1*	prostaglandin-endoperoxide synthase 1	0.179	Cytoplasm	enzyme
ENSG00000207217	N/A	unidentified	0.178	N/A	N/A
ENSG00000210140	N/A	unidentified	0.172	N/A	N/A
ENSG00000019991	*HGF*	hepatocyte growth factor (hepapoietin A; scatter factor)	0.153	Extracellular Space	growth factor
ENSG00000261519	N/A	unidentified	0.153	N/A	N/A
ENSG00000228360	*UBN2*	ubinuclein 2	0.149	Nucleus	other
ENSG00000265764	N/A	unidentified	0.146	N/A	N/A
ENSG00000134321	*RSAD2*	radical S-adenosyl methionine domain containing 2	0.138	Cytoplasm	enzyme
ENSG00000227205	N/A	unidentified	0.110	N/A	N/A
ENSG00000217624	N/A	unidentified	0.103	N/A	N/A
ENSG00000201533	N/A	unidentified	0.078	N/A	N/A
ENSG00000202240	N/A	unidentified	0.075	N/A	N/A
ENSG00000226970	N/A	unidentified	0.069	N/A	N/A
ENSG00000199753	*SNORD104*	small nucleolar RNA, C/D box 104	0.063	unknown	other
ENSG00000207168	*SNORA15*	small nucleolar RNA, H/ACA box 15	0.059	unknown	other
ENSG00000206795	N/A	unidentified	0.057	N/A	N/A
ENSG00000255633	N/A	unidentified	0.047	N/A	N/A
ENSG00000118971	*CCND2*	cyclin D2	0.045	Nucleus	other
ENSG00000066056	*TIE1*	tyrosine kinase with immunoglobulin-like and EGF-like domains 1	0.042	Plasma Membrane	kinase
ENSG00000199455	*RNA5SP191*	RNA, 5S ribosomal pseudogene 191	0.028	unknown	other
ENSG00000251718	N/A	unidentified	0.015	N/A	N/A
ENSG00000201766	N/A	unidentified	0.011	N/A	N/A
ENSG00000199461	N/A	unidentified	0.009	N/A	N/A
ENSG00000200496	N/A	unidentified	0.008	N/A	N/A
ENSG00000201800	N/A	unidentified	0.006	N/A	N/A
ENSG00000257231	N/A	unidentified	0.006	N/A	N/A
ENSG00000200107	N/A	unidentified	0.005	N/A	N/A
ENSG00000265802	N/A	unidentified	0.002	N/A	N/A
ENSG00000263911	N/A	unidentified	0.002	N/A	N/A
